# Effects of Sodium Butyrate and Organic Zinc Supplementation on Performance, Mineral Metabolism, and Intestinal Health of Dairy Calves

**DOI:** 10.3390/ani16020230

**Published:** 2026-01-13

**Authors:** Mellory M. Martins, Larissa S. Gheller, Bruna L. de Noronha, Gabrielly A. Cassiano, Mariana B. Figueiredo, Caroline M. Meira, Flávia F. Simili, Márcia S. V. Salles, Arlindo Saran Netto

**Affiliations:** 1 Department of Animal Science, College of Animal Science and Food Engineering, University of São Paulo, Pirassununga 13635-900, SP, Brazil; mellory.martins@usp.br (M.M.M.); bruna.noronha@usp.br (B.L.d.N.); saranetto@usp.br (A.S.N.); 2 Animal Science Institute (IZ), Ribeirão Preto 14030-640, SP, Brazil; lsgheller@gmail.com (L.S.G.); ga.cassiano@unesp.br (G.A.C.); marianabenettifigueiredo@hotmail.com (M.B.F.); flavia.simili@sp.gov.br (F.F.S.); 3 Department of Veterinary Medicine, College of Animal Science and Food Engineering, University of São Paulo, Pirassununga 13635-900, SP, Brazil

**Keywords:** calf nutrition, dairy calves, diarrhea incidence, microelement, weight gain

## Abstract

The objective of this study was to evaluate the effects of sodium butyrate and organic zinc supplementation, alone or combined, on performance, zinc metabolism, blood parameters, and gut health in Holstein calves highly challenged by heat and diarrhea during the pre-weaning and weaning periods. Forty-eight male calves were assigned to one of four treatments: control (CON), SB (3 g/kg of sodium butyrate in dry matter [DM]), OZn (262 mg/kg of organic zinc in DM), or SBOZn (3 g/kg of sodium butyrate and 262 mg/kg of organic zinc in DM). Calves were monitored from days 7 to 63 for feed intake, weight gain, body morphometry, fecal score, and blood parameters. Zinc balance was evaluated from days 45 to 49, and 24 calves were slaughtered on day 64 for jejunal sampling to assess tight junction gene expression. Diarrhea incidence was high (>90%) across groups. Fecal scores varied over time but did not differ between treatments. The OZn and SBOZn groups had higher Zn intake, with greater absorption and retention of the mineral compared to the CON and SB groups. Additionally, the OZn group tended to have higher serum Zn concentrations. SB and OZn, separately or combined, had limited effects and did not consistently improve the performance or health of calves highly challenged during pre-weaning and weaning.

## Simple Summary

The rearing phase is a challenging period in dairy farming. It involves environmental, health, and physiological stressors that can affect calf growth and well-being. Nutritional strategies such as sodium butyrate and organic zinc supplementation have been proposed to mitigate these effects. Therefore, this study evaluated the effects of sodium butyrate and organic zinc supplementation, alone or in combination, on the performance, zinc balance, intestinal health, and oxidative stress markers of dairy calves under heat stress and high sanitary challenge. Forty-eight male calves were assigned to one of four groups: control group, group supplemented with sodium butyrate, group supplemented with organic zinc, and group supplemented with both. Calves were monitored for feed intake, weight gain, body measurements, and fecal score. Zinc absorption was also evaluated, and some calves were slaughtered to assess intestinal permeability. The incidence of diarrhea was high in all groups, and supplementation with sodium butyrate and/or organic zinc did not reduce its occurrence. There were significant increases in Zn absorption and retention. However, the additives, whether given alone or together, had limited effects and did not improve calf growth or health under high environmental and sanitary challenges.

## 1. Introduction

The rearing phase is one of the most critical periods in dairy farming, as it involves multiple environmental, health, and physiological stressors that can compromise the calves’ welfare and performance [1]. Among the main challenges are enteric diseases, particularly neonatal diarrhea [2]. This condition leads to high morbidity and mortality rates, dehydration, low performance, and elevated treatment costs [3,4,5,6]. These effects can be intensified in systems under high sanitary pressure, such as raising calves in a collective system, and heat stress, resulting in reduced feed intake [7] and impaired immune responses [8].

Given the aforementioned challenges, nutritional strategies such as zinc and sodium butyrate supplementation have been explored to mitigate negative impacts on calf health and performance [9,10,11,12,13]. Zinc is an essential trace element that plays a key role in maintaining epithelial integrity, supporting cell proliferation, and modulating immune functions [14]. Zinc deficiency in livestock is linked to reductions in growth performance, impairments in reproductive function and greater susceptibility to diseases [15]. In contrast, supplementation with zinc in neonates or young calves has been associated with reduced duration and severity of diarrhea, improved weight gain and feed efficiency, and enhanced intestinal barrier integrity [9,16,17,18]. Furthermore, milk-based supplementation has been associated with increased serum Zn concentrations and reduced days with high fecal scores, suggesting improved mineral absorption and intestinal health [9,11].

Sodium butyrate has been associated with improved gut health, immunomodulatory effects, increased average daily gain, and reduced incidence of diarrhea [13,19,20,21,22]. These benefits occur because butyrate stimulates rumen papillae development, promotes epithelial cell proliferation, enhances rumen fermentation and serves as an important energy substrate for intestinal cells [13,23]. Additionally, its regulatory effects are mediated through short-chain fatty acid-sensing G-protein-coupled receptors and the inhibition of histone deacetylase, thereby modulating inflammatory and metabolic signaling pathways [24,25]. Studies indicate that the delivery method influences its effects [26]. When added to milk replacer, sodium butyrate has been associated with higher dry matter intake, whereas inclusion in the starter has been related to greater final body weight (BW) and a tendency for higher average daily gain [26].

Despite these findings, most studies evaluating the effects of zinc and sodium butyrate supplementation did not describe the environmental and sanitary conditions in which the studies were conducted. Variability in management, exposure to pathogens and heat stress can influence the biological response to supplementation, which highlights the importance of testing these strategies under more challenging conditions. Taken together, zinc and sodium butyrate may have complementary effects under adverse conditions. Zinc primarily supports antioxidant defense and epithelial integrity, while sodium butyrate promotes the energy supply of the epithelium, intestinal development, and anti-inflammatory signaling. This combination could theoretically increase the intestinal resilience of calves exposed to heat stress and health challenges.

The objective of this study was to evaluate the effects of supplementing organic zinc and sodium butyrate, alone or in combination, on the performance, zinc balance, intestinal health, and oxidative stress markers of dairy calves under heat stress and high sanitary challenge. We hypothesized that zinc supplementation would increase systemic mineral availability and support immune function, while sodium butyrate would improve intestinal integrity and performance. We expected that the combination of both additives could potentiate their individual effects, promoting better intestinal integrity and performance in calves.

## 2. Materials and Methods

**Figure 1:**
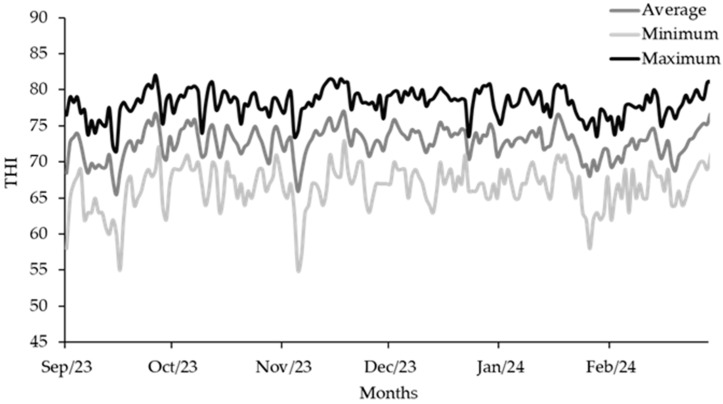
Average, minimum, and maximum temperature–humidity index (THI) during the experimental period, calculated according Mader et al. [27].

This study was conducted between September 2023 and February 2024 at the experimental farm of the São Paulo Agency for Agribusiness Technology, Institute of Animal Science, located in Ribeirão Preto (São Paulo, SP, Brazil). The temperature–humidity index (THI) was monitored throughout the experimental period and calculated according to the equation proposed by Mader et al. [27], based on temperature and relative humidity data obtained from a weather station located near the experimental farm. The average, minimum, and maximum THI values throughout the experimental period are shown in Figure 1.

### 2.1. Experimental Design, Treatments, and Diets

Forty-eight Holstein male calves, with an initial BW of 40.93 ± 4.541 kg, were obtained from a commercial dairy farm located approximately 130 km from the experimental facility. The dams were fed with a total mixed ration consisting of corn silage and Tifton hay as forage sources, as well as a concentrate based on corn, soybean meal, and cottonseed meal. The herd consisted exclusively of Holstein cows. No information was collected on the dams’ health status or routine health-monitoring indices, such as antibody levels. Calves were separated from their dams immediately after birth, had their navels disinfected with 10% iodine tincture, and were weighed. Within 2 h of birth, each calf received 447 g of bovine colostrum replacer (Premolac Zn; Zinpro Corp., Eden Prairie, MN, USA) diluted in 2 L of cow’s transition milk at 39 °C, providing ≥50 mg/mL IgG, as recommended by Godden [28]. This colostrum replacer has demonstrated efficacy in IgG absorption in calves [29] and is commercially marketed as compliant with USDA—Center for Veterinary Biologic standards regarding minimum IgG content, purity, potency, and manufacturing quality [30].

Additionally, a blood sample was collected on the third day of the calf’s life by jugular vein puncture into a vacutainer tube with clot activator. The samples were centrifuged at 2000× *g* for 20 min. The obtained serum was transferred to microtubes and stored at −20 °C for subsequent IgG concentration analysis using a radial immunodiffusion kit (Triple J Farms, Bellingham, WA, USA). The calves remained on the commercial farm until the third day of life, when they were transported to the experimental farm.

Calves were divided into blocks according to month of birth, and randomly assigned to one of the following treatments: (a) control (CON, basal diet with no supplementation); (b) sodium butyrate (SB, supplementation of 3 g/kg DM of sodium butyrate, Adimix Easy, Socil Animal Nutrition, Descalvado, SP, Brazil); (c) organic zinc (OZn), supplementation of 262 mg/kg DM of organic zinc, B-Traxim, (Neovia Animal Nutrition and Health, Apucarana, PR, Brazil); and (d) sodium butyrate plus organic zinc [SBOZn, 3 g/kg DM of sodium butyrate (Adimix Easy, Socil Animal Nutrition, Descalvado, SP, Brazil) and 262 mg/kg DM of organic zinc, (B-Traxim, Neovia Animal Nutrition and Health, Apucarana, PR, Brazil)]. Sodium butyrate was incorporated directly into the starter during preparation, whereas organic zinc was administered orally daily using a 10 mL syringe. The sodium butyrate inclusion level (3 g/kg DM; ~0.3%) corresponds to doses widely used in calf studies [13,20] and aligns with NASEM (2021) [31]. For organic zinc, a higher inclusion level than that used in more recent experimental studies [15,17,32] was adopted based on the assumption that an increased Zn supply could be more effective under the highly challenging sanitary conditions of this experiment.

The calves were housed in two collective paddocks with a total area of 576 m^2^, containing identical structure, with 48 m^2^ of shade provided by a shelter with a roof and sand bedding. Both paddocks were constructed with the same dimensions, shade area, bedding type, and layout to ensure homogeneous microclimatic conditions across groups. The paddocks were equipped with automatic feeding systems for providing milk replacer (DeLaval^®^ automatic feeder, Tumba, Sweden), starter, and water (Intergado Ltda., Contagem, MG, Brazil). The calves were individually identified using tags, allowing controlled feed access. Commercial milk replacer (20.05% crude protein and 14.12% ether extract; Amamenta Premium^®^, Neovia Animal Nutrition and Health, Apucarana, PR, Brazil) was available 24-h a day, with a dilution of 140 g solids/L. The milk-feeding protocol consisted of 6 L/d of milk replacer from d 7 to d 49, 4 L/d from d 50 to d 56, 2 L/d from d 57 to d 63, and withdrawal of milk replacer from d 64 onwards. The starter feed (Nutris Inicial^®^, Socil Animal Nutrition, Descalvado, SP, Brazil) was available 24 h a day, initially offered at a rate of 100 g per calf per day and subsequently adjusted based on the individual consumption of each calf. The starter was offered ad libitum throughout the experimental period. For organic zinc, a higher inclusion level than that used in more recent experimental studies [15,17,33] was selected based on the hypothesis that an elevated Zn supply could better support mineral balance and immune-related responses under the high sanitary and heat stress conditions of this experiment.

**Figure 2:**
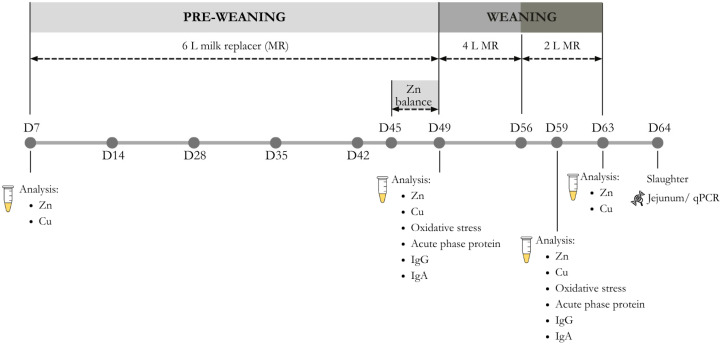
Experimental and sampling schedule, illustrating key management practices and sampling points during the pre-weaning and weaning periods.

The chemical composition of the milk replacer and the starter is shown in Table 1. Figure 2 shows the experimental schedule, indicating the milk feeding regimen and the main analyses performed during the study.

### 2.2. Feed Intake and Performance

Milk replacer, starter, and water consumption were monitored and recorded daily using automatic feeders (DeLaval^®^ feeder for the milk replacer and Intergado Ltda. feeders and drinkers for the starter and water). Milk replacer was offered at a set amount based on the calf’s age, while the amount of starter was adjusted individually according to daily intake. Milk replacer and starter samples were collected every two weeks for chemical analysis to determine nutrient intake.

Calves were weighed weekly using an electronic scale. Height at the withers, hip width, and body length were measured on days 7, 21, 35, 42, 56, and 63 (corresponding to weeks 1, 3, 5, 7, 8, and 9). Withers height was measured using a hipometer to record the distance from the ground to the highest point of the withers. Hip width was determined using a hipometer to measure the distance between the ischia. Body length was measured from the scapula to the beginning of the tail insertion with a tape measure. All body measurements were taken with the animal standing on a flat surface, with all four legs touching the ground. All body measurements were performed by the same evaluator.

### 2.3. Zinc Balance

A zinc balance trial was conducted starting on day 45 for 96 consecutive hours. Twenty-four animals (six per treatment) were kept in metabolic cages with rubber-coated flooring. All feces and urine were collected during the trial. The feces were collected in bags, weighed, and sampled at 10% of the daily total. Urine was collected in plastic buckets adapted to the cages. The volume was recorded, and samples were taken at 5% of the daily total. Milk replacer, starter, and water intake were recorded daily, and samples of both the milk replacer and the starter were collected. At the end of 96 h, the feces, urine, and feed samples were homogenized, and an aliquot was frozen at −20 °C for further analysis.

### 2.4. Fecal Score and Incidence of Diarrhea

The fecal score was assessed and recorded daily according to stool consistency and general appearance, as proposed by McGuirk [34]. Stools were classified as: (0) normal and firm, (1) pasty but generally healthy, (2) liquid, and (3) watery. Calves were considered to have diarrhea if they had a score of ≥2 for two consecutive days [35]. All calves with a fecal score ≥ 2 received a total of 4 L/day of oral electrolyte solution (Alta LYTE^®^, Alta Brasil, Patos de Minas, MG, Brazil), divided into two daily administrations of 2 L each. Electrolytes were offered after the milk-feeding period, with an average interval of approximately two hours between milk replacer intake and electrolyte administration. Animals were monitored using the Foster Technik^®^ application (Foster Technik, Engen, Germany), and electrolytes were first offered via bottle; if voluntary intake did not occur, an esophageal tube was used. Calves were considered healthy when they had a score of ≤1 for two consecutive days [36].

Fecal samples were collected from 64% of calves (*n* = 31; *n* = 7 in group CON, *n* = 7 in group SB, *n* = 7 in group OZn, and *n* = 10 in group SBOZn) during their first episode of diarrhea for pathogens identification. All samples analyzed (100%; *n* = 31) tested positive for *Cryptosporidium* spp. oocysts at varying quantitative levels. No other pathogens were identified.

The samples were obtained directly from the rectum using disposable plastic gloves, properly labeled, kept refrigerated (2 to 8 °C), and sent to a microbiological laboratory for analysis. The tests included the detection of *Cryptosporidium* spp. (modified Kinyoun staining technique), rotavirus (qualitative immunochromatographic assay), and OPG and EPG counts (oocysts and eggs per gram, respectively). Additionally, cultures were performed for *Salmonella* spp. (selective media and polyvalent serology) and *Escherichia coli* (differential culture medium and serotyping of pathogenic strains A, B, and C by slide agglutination).

The frequency of each diarrhea score was calculated using the following equation:

$$Frequency\;\left(\%\right)=\;\frac{\left(number\;of\;days\;in\;the\;score\;\times 100\right)}{total\;number\;of\;evaluated\;days}$$

### 2.5. Tight Junction Protein Gene Expressions Detected by Real-Time PCR

#### 2.5.1. RNA Extraction and cDNA Synthesis

Twenty-four calves (six from each treatment) were humanely slaughtered on d 64 using a non-penetrating captive bolt pistol for stunning, followed immediately by exsanguination. All procedures were performed by trained personnel in accordance with the Brazilian Guide of Best Practices for the Euthanasia of Animals—Recommended Concepts and Procedures (https://www.gov.br/agricultura/pt-br/assuntos/producao-animal/arquivos-publicacoes-bem-estar-animal/guia-brasileiro-de-boas-praticas-para-a-eutanasia-em-animais.pdf, accessed on 5 January 2026) and were approved by the Animal Use Ethics Committee of the Animal Science Institute (protocol n° 365-2022).

Jejunal fragments measuring approximately 1 cm^3^ were collected aseptically. Total RNA was extracted using TRIzol^®^ reagent (Invitrogen, Waltham, MA, USA) according to the manufacturer’s instructions. RNA concentration and purity were determined using a NanoDrop 2000 spectrophotometer (Thermo Scientific, Waltham, MA, USA) based on the 260/280 nm absorbance ratio, while integrity was verified by electrophoresis on 1.5% agarose gels. All RNA samples were treated with DNase I (Invitrogen, USA) to remove residual genomic DNA and then stored at −80 °C until use. Reverse transcription was performed with the High-Capacity cDNA Reverse Transcription Kit (Applied Biosystems, Waltham, MA, USA), following the manufacturer’s protocol.

#### 2.5.2. qPCR Analysis

Primers were designed based on Ma et al., [17] and underwent a standardization process to ensure satisfactory amplification efficiency. Efficiency was determined using standard curve analysis with the equation E = (10^−1/slope^ − 1) × 100, and only primers with efficiencies between 90–110% were accepted.

Quantitative PCR reactions were carried out in duplicate in a final volume of 10 µL, consisting of 5 µL PowerUp™ SYBR^®^ Green Master Mix (Applied Biosystems), 200 nM of each primer, 25 ng of cDNA, and ultrapure water. Amplifications were performed in a CFX96 Touch Real-Time PCR Detection System (Bio-Rad, Hercules, CA, USA). No-template controls were included to check for contamination.

Following each amplification, melting curve analysis (55–95 °C, 0.5 °C/30 s) was performed to confirm specificity and the absence of primer-dimer formation. The quantification cycle (Cq) values of target genes were normalized (ΔCq) against the geometric mean of the endogenous reference genes β-actin. Relative expression levels were calculated using the 2^−ΔCt^ method [37].

### 2.6. Blood Sampling

Blood samples (~10 mL) were collected via jugular venipuncture into clot activator vacutainer tubes to determine serum zinc and copper concentrations at 7, 49, and 63 d of age. Additional samples for acute phase proteins and oxidative stress markers were obtained on d 49 and 59, coinciding with the weaning process, a natural stressor for calves. Immediately after collection, samples were centrifuged at 2000× *g* for 15 min, and the obtained serum was aliquoted into microtubes and stored at −20 °C until analysis.

### 2.7. Analytical Methodologies

The milk replacer and starter samples were pre-dried in a forced ventilation oven at 55 °C for 72 h and then processed in a Wiley mill (MA340, Marconi, Piracicaba, SP, Brazil) using a 1 mm sieve. The samples were then analyzed for dry matter (DM) content (method 930.15; AOAC [38]), crude protein (CP) (N × 6.25; method 984.13; AOAC [38]), ether extract (EE) (method 920.39; AOAC [38]), ash (method 942.05; AOAC [38]), and neutral detergent fiber (NDF) using alpha-amylase without adding sodium sulfite [39]. Fecal samples underwent the same analyses.

The concentrations of zinc and copper in feed, serum, feces, and urine samples were determined using inductively coupled plasma optical emission spectrometry (ICP-OES), as described in [40].

Oxidative stress markers were determined in serum samples using commercial kits (Cayman Chemical, Ann Arbor, MI, USA) specifically designed for superoxide dismutase (SOD, #706002) and total antioxidant activity (ASD, #709001), in accordance with the manufacturer’s instructions.

Acute phase proteins (ceruloplasmin, transferrin, albumin, haptoglobin) and immunoglobulins (Immunoglobulin A, immunoglobulin G) concentrations were measured in serum samples. Serum protein was determined by the biuret method using a commercial kit (Total Protein Ref. 99–100; Labtest, Lagoa Santa, MG, Brazil). The separation of protein fractions was performed by acrylamide gel electrophoresis with sodium dodecyl sulfate (SDSPAGE), according to Laemmli [41]. After fractionation, the gel was stained with Coomassie blue solution (50.0% methanol, 40.0% water, 9.75% glacial acetic acid, and 0.25% Coomassie blue) for 10 min. The gel was then placed in a 7.0% acetic acid solution to remove excess dye until the protein fractions were clear. Acute phase protein concentrations were determined using a computerized densitometer.

### 2.8. Statistical Analysis

The data were analyzed using SAS software, version 9.4 (SAS Institute Inc., Cary, NC, USA). Prior to analysis, data on milk replacer, starter, water, and fecal score intake were grouped by week. The normality of the residuals and the homogeneity of the variances were verified using PROC UNIVARIATE.

Feed intake and performance variables, blood variables, and fecal scores were analyzed as repeated measures over time using PROC MIXED according to the following model:

$$Y_{ijk}=\mu +B_{i}+T_{j}+D_{k}+{\left(T\times D\right)}_{jk}+e_{ijk}$$

where $Y_{ijk}$, is the dependent variable; μ is the overall mean; $B_{i}$ is the random block effect; $T_{j}$ is the fixed treatment effect; $D_{k}$ is the fixed effect of sampling day; ${\left(T\times D\right)}_{jk}$ is the interaction between treatment and sampling day; and $e_{ijk}$ is the residual error. For data collected at equal intervals, a first-order autoregressive [AR(1)] covariance structure was used, which assumes decreasing correlations over time. For data collected at different time intervals, the spatial power [SP(POW)] covariance structure was used, which is appropriate for this type of temporal distribution.

The data on nutrient digestibility, zinc balance, and tight junction protein gene expression were analyzed using PROC MIXED according to the following model:

$$Y_{ij}=\mu +B_{i}+T_{j}+e_{ij}$$

where $Y_{ij}$, is the dependent variable; μ is the overall mean; $B_{i}$ is the random block effect; $T_{j}$ is the fixed treatment effect; and $e_{ij}$ is the residual error.

Covariates were tested according to the nature of each dependent variable and included in the model when *p* ≤ 0.05. Feed intake variables were tested using serum IgG concentration and birth weight; performance variables were tested using measurements from the first week of life; and serum Zn and Cu concentrations were tested using values obtained at the D7 sampling.

Variables with a binomial distribution, such as the incidence of diarrhea, were analyzed using Pearson’s chi-square test to evaluate the association between groups. If 50% or more of the cells had expected frequencies of less than 5, the exact test was used instead.

Values are presented as least squares mean. For all results, differences were considered significant if *p* ≤ 0.05 and a tendency if *p* > 0.05 and ≤0.10. The differences between treatments were estimated by Tukey’s range test.

## 3. Results

No differences were observed between the groups in serum IgG concentration on the third day of life (CON: 29.0 ± 2.77; SB: 27.8 ± 2.65; OZn: 27.1 ± 2.77; SBOZn: 30.4 ± 2.77 g/L; *p* = 0.840), confirming that all calves were properly colostrum-fed. Supplementation with SB, OZn, or their combination did not affect the DMI, starter or milk replacer intake, water intake, daily weight gain, feed efficiency or body measurements of calves during the pre-weaning and weaning periods (Table 2; *p* ≥ 0.126). No interactions between treatment and time were found for the variables evaluated (*p* ≥ 0.286).

Calves in the OZn and SBOZn groups had a higher Zn intake (Table 3; *p* = 0.002) than those in the CON and SB groups. Apparent Zn absorption was higher (*p* = 0.019) in the OZn and SBOZn groups than in the CON and SB groups. Similarly, Zn increased (*p* = 0.017) in the OZn and SBOZn groups compared to the CON and SB groups. There were no differences in fecal, urinary, or total Zn excretion (*p* ≥ 0.545) or in the percentage of Zn absorbed or excreted (*p* = 0.746).

Diarrhea incidence was high in all groups (Table 4) and no differences were observed among the treatments (*p* = 0.382). The fecal score did not differ among treatments (*p* = 0.293) but varied over time (*p* < 0.0001), with a peak in the second week of life followed by a gradual decrease until weaning (Figure S1). A similar pattern was observed in the frequency of scores 2 and 3. Although these frequencies did not differ between treatments, they were concentrated in the second week and decreased in subsequent weeks (Figure S2). In addition, supplementation with SB, OZn, or their combination did not alter (Table 5; *p* ≥ 0.135) the gene expression of tight junction proteins in the jejunum of calves.

Regarding serum Zn concentrations, there was a tendency for an increase among the treatments (Table 6; *p* = 0.071). Higher values were observed in the OZn group than in the CON and SB groups. The SBOZn group presented intermediate values and did not differ from the other groups. There were no differences among treatments for serum Cu (*p* = 0.256), nor were there any interactions between treatment and time (*p* ≥ 0.648).

During the weaning period, no differences were observed between treatments (Table 7; *p* ≥ 0.108) in serum concentrations of albumin, haptoglobin, IgA, transferrin, or IgG. The ceruloplasmin concentration was higher in the SBOZn group than in the CON and OZn groups (*p* = 0.015). There was a tendency for SOD concentration to decrease (*p* = 0.087) in the OZn and SBOZn groups compared to the CON and SB groups. ASD was not influenced by the treatments (*p* = 0.219), and no significant interactions between treatment and time were observed.

## 4. Discussion

This study evaluated the effects of supplementing calves with SB and OZn, alone or in combination (SBOZn), during the pre-weaning period under conditions of health challenges and heat stress. Regarding the health challenge, most calves showed signs of diarrhea, as indicated by fecal scores, which were more pronounced during the first two weeks of the preweaning period and milder in the subsequent weeks.

This high incidence may have been due to the collective housing system, where pathogen transmission is greater [42]. In addition, the pathogen involved, *Cryptosporidium* spp., is highly resistant and widely disseminated in the environment, which may have intensified the contamination [43,44]. The elevated THI most probably also contributed to the increased occurrence of diarrhea, as the animals remained under heat stress for most of the experimental period, a condition known to reduce immunocompetence and increase susceptibility to enteric infections [45,46].

As noted, the animals spent most of the experimental period under heat stress conditions. Wang et al. [47] reported THI thresholds for heat stress in dairy cattle, defining moderate heat stress as ranging from 72 to 80, severe from 80 to 90, and emergency conditions above 90. Other authors, investigating THI amplitude in preweaning dairy calves, reported that calves should remain within THI values of 65 to 69; above 69, physiological and metabolic alterations may occur [48]. Kovács et al. [49], in a study aimed at determining the upper critical THI limits in Holstein calves, inferred that the welfare of young calves may be compromised above a THI of 78, and that calves experience significant heat stress above a THI of 88. It is noteworthy that there is still no consensus in the literature regarding the optimal THI range for these animals; however, it can be considered that the calves in the present study remained, on average, most of the time at THI values above 70 but below those classified as severe.

A low initial starter intake (0.256–0.324 kg/day) was observed, and the average DMI of the supplemented calves throughout the experiment was 0.928 kg. NASEM [31] recommends a DMI ranging from 0.93 to 1.10 kg/day for calves weighing 50 to 70 kg of body weight, with a daily weight gain of 400 g. The lower intake observed can be partially explained by environmental (such as elevated THI) and sanitary conditions (*Cryptosporidium* spp.). In our study, the THI had a median of 73, which is higher than the 65-threshold considered sufficient to induce physiological heat stress responses in calves [48]. Previous studies have reported that heat stress can significantly increase respiratory rate [50], reduce feed intake [7] and compromise calf performance [51,52]. High temperatures activate the hypothalamic thermoregulatory center, which inhibits the appetite center, as an adaptive mechanism to reduce metabolic heat production, resulting in hypophagia [53,54]. Additionally, heat stress is associated with hormonal changes, such as increased insulin and reduced thyroid hormones, which suppress appetite and redirect energy from growth to maintaining homeothermy [52,54], although this type of assessment was outside the scope of our study. In addition to the effects of heat stress, sanitary challenges also played an important role in limiting calf intake and performance. Clinical cases of diarrhea are known to reduce dry matter intake, compromise feed efficiency, and consequently impair calf performance [55]. Even with sodium butyrate supplementation, low intake may have limited the effective intake of sodium butyrate, which may explain the lack of effect on performance. Furthermore, the addition of sodium butyrate to the calf’s initial feed may have led to a decrease in intake due to its strong odor and taste, as calves are highly sensitive to feed flavor [56].

Previous studies have reported greater weight gain in calves supplemented with 0.3% butyrate in the starter (the same dose used in our study), with a starter intake of 0.86 kg/day [22]. Furthermore, Górka et al. [26] observed that the method of supplying butyrate can influence calf performance; increased dry matter intake was observed when butyrate was included in a protected form in the starter (0.6%), while greater weight gain was observed when butyrate was added to the milk replacer (0.3%). Wanat et al. [56] also highlighted that the effects of butyrate depend on both starter composition and administration strategy. On the other hand, Belli et al. [57] provided 4 g/day of sodium butyrate directly in milk replacer and observed no effects on feed efficiency or average daily gain in calves, results that are similar to those found in the present study. Alternative approaches, such as the use of microencapsulated or protected butyrate [27], incorporation of flavorings to improve palatability [55], or administration through liquid diets [22], may enhance butyrate intake and bioavailability, particularly under conditions of low starter intake; however, these strategies were not applied in this study.

Zn supplementation resulted in increased Zn intake and absorption/retention in the OZn and SBOZn groups, and all groups showed serum zinc concentrations above the deficiency threshold (<0.05 mg/L). This was reflected in higher serum Zn concentrations in the OZn group compared to the CON and SB groups. There was no change in serum Cu concentrations, and the values remained within the physiological reference range (above 0.05 mg/L; NASEM, 2021 [31]), indicating absence of Cu deficiency, maintaining the Cu:Zn balance and avoiding the antagonistic effects that can occur at high Zn levels [58,59]. These findings indicate that OZn supplementation increases systemic Zn availability, corroborating previous results [9,10,11,60] and reinforcing the efficiency of intestinal absorption. Zn supplementation is therefore an effective way to increase serum Zn accumulation [61,62].

Despite the increased availability of Zn, no differences in the incidence of diarrhea were observed. However, studies indicate that Zn supplementation can reduce the incidence of diarrhea and improve calf performance [9,10,16,17,60]. In our study, the high incidence of diarrhea (91–100%), associated with the presence of *Cryptosporidium* spp., probably limited the physiological response of calves to Zn supplementation. Thus, the pathogen involved may partly explain the differences in responses observed among studies. In the present study, the predominant presence of *Cryptosporidium* spp. may have influenced the physiological and immunological responses to supplementation, given that different pathogens activate different immune pathways [63,64] and may respond differently to Zn availability. During bacterial infections, the host reduces systemic Zn availability to limit microbial growth [65]. Nonetheless, *Cryptosporidium* spp. is an intracellular, extracytoplasmic parasite that develops in an intermediate location within the intestinal epithelium, enabling access to host nutrients, such as zinc, while partially avoiding immunological restriction mechanisms [66]. Therefore, the nature and location of the pathogen can significantly influence the host’s response to mineral supplementation, highlighting the need for studies focusing on the specific interactions between Zn, the host and *Cryptosporidium* spp. Similar to our study, Feldmann et al. [11] observed an increase in serum Zn concentrations in supplemented calves. However, although they reported clinical improvement with a shorter duration of diarrhea and faster recovery, Zn supplementation did not affect microbiological cure and was associated with greater fecal elimination of *C. parvum* [11].

Supplementation with SB alone or in combination with Zn did not affect the incidence of diarrhea. There have been few studies that have specifically evaluated the effect of SB on the incidence of diarrhea, particularly when it is offered via starter [13]. Most research has focused on gastrointestinal development, performance, feed efficiency, and modulation of the immune response [20,23,56,67]. Górka et al. [26] reported a trend towards fewer days of electrolyte therapy and improved fecal consistency in calves receiving 0.3% sodium butyrate via milk replacer. However, Wanat et al. [56] observed an increase in days with diarrhea as the supplementation level increased (0.3%, 0.6%, and 0.9%). Furthermore, Nicola et al. [68] demonstrated that supplementing whole milk with 4 g/day of SB reduced the number of days with abnormal stools and decreased the occurrence and severity of diarrhea. Given this, supplementation via starter, combined with low average consumption and high sanitary pressure in our study, may have limited effective intake and consequently the expected physiological response.

The variation in fecal scores over the weeks, peaking in the second week of life, is consistent with data indicating that the first two weeks are the period of greatest susceptibility to neonatal diarrhea [4,6]. In our study, fecal scores greater than 2 were classified as diarrhea, and the increase observed during this period reflects this clinical condition. This phase coincides with the immaturity of the intestinal epithelium and immune system, resulting in increased permeability and fluid loss [69,70]. Additionally, the observed high prevalence is consistent with field data indicating that mortality associated with digestive diseases predominantly occurs up to 24 days of age [5]. Supplementation with SB, OZn and SBOZn did not alter the expression of junction proteins in the jejunum, even though doses of 80 mg Zn/day have been linked to increased expression of these proteins and enhanced intestinal barrier integrity [17]. The lack of effect observed in our study may be related to the health challenges faced and the high incidence of diarrhea, which may have obscured the positive effects of Zn.

Regarding oxidative stress and acute phase markers, an increase in serum ceruloplasmin was observed in the SBOZn group, along with a tendency towards reduced superoxide dismutase (SOD) activity in the OZn and SBOZn groups. These findings suggest that supplementation may influence antioxidant mechanisms, even when there are no effects on performance or immunological parameters. Ceruloplasmin is a multicopper oxidase that has both antioxidant and acute-phase protein functions and is synthesized mainly in the liver [71,72,73]. Although it is often associated with inflammatory response in ruminants [73,74,75], an increase in ceruloplasmin does not necessarily indicate an acute phase response. Previous studies have demonstrated elevated ceruloplasmin in small ruminants, accompanied by increased concentrations of haptoglobin and fibrinogen [74,75]. In a study by Abdelsattar et al. [74], the simultaneous elevation of ceruloplasmin and haptoglobin in the group receiving the highest butyrate dose was interpreted as an acute-phase inflammatory response. However, in our study, an increase in ceruloplasmin occurred without an increase in haptoglobin, recognized as one of the main markers of inflammation in cattle [76], or a reduction in total antioxidant capacity, and there was no change in the incidence of diarrhea. This suggests that ceruloplasmin predominantly plays an antioxidant role rather than an inflammatory one.

Zn supplementation may have contributed to this modulation, as Zn regulates Cu/Zn-SOD activity in a dose-dependent manner [33], and, at higher levels, stimulates the synthesis of metallothioneins (MTs), non-enzymatic antioxidant proteins [77]. MTs protect cells against the cytotoxic effects of reactive oxygen species by releasing Zn during oxidative processes, thereby activating dependent enzymes such as SOD [77,78]. Higher MT expression may reduce the need for high enzymatic activity, which could explain the lower SOD values observed. The observed pattern of higher ceruloplasmin and a tendency towards lower SOD activity may indicate a predominance of non-enzymatic pathways over a typical response to oxidative stress or inflammation. As serum Cu concentration was not altered, Cu-Zn antagonism can be ruled out as an explanation for the reduction in SOD.

Although supplementation with SB and ZnO increased zinc status, this effect did not translate into improvements in health or calf performance because the overall conditions of the experiment involved a combination of a high sanitary challenge, predominantly caused by *Cryptosporidium* spp., persistent heat stress, and low starter intake. Furthermore, even though supplementation was provided throughout the experimental period, it cannot be ruled out that the amount of sodium butyrate consumed was insufficient to provide adequate support during critical periods of intestinal challenge and heat stress. This study did not evaluate possible interactions with other nutrients involved in antioxidant defense and immune function, such as vitamin E or selenium, which may have influenced the response to supplementation. Together, these factors likely limited sodium butyrate intake and availability and masked physiological responses associated with zinc supplementation. From a practical standpoint, these results reinforce that feed additives, in this case, zinc and sodium butyrate, should be regarded as supportive rather than corrective strategies. Their effectiveness depends on appropriate management conditions and may be limited under scenarios of intense challenge.

## 5. Conclusions

Supplementation with sodium butyrate and organic zinc, alone or in combination, increased systemic zinc availability and modulated antioxidant responses in pre-weaning calves exposed to heat stress and high sanitary challenge. However, these changes did not lead to improvements in growth performance, intestinal barrier function, or overall health status. These findings suggest that the effectiveness of sodium butyrate and zinc supplementation depends on adequate environmental and management conditions. Further studies are warranted to elucidate pathogen-specific immune and oxidative responses in challenged calves and to optimize supplementation strategies under different stress conditions.

## Figures and Tables

**Table 1:** Chemical composition of the milk replacer and starter fed to calves.

Item	Milk Replacer ^1^	Starter ^2^
Chemical composition (g/kg)		
Dry matter	951.4	917.4
Organic matter	912.9	849.0
Crude protein	200.5	163.4
Ether extract	141.2	29.10
Neutral detergent fiber	13.00	482.1

^1^ Mineral and vitamin composition: Ca 1.2%; P 0.7%; Na 0.6%; Fe 79.95 mg/kg; Cu 10.5 mg/kg; Mn 115.1 mg/kg; Zn 100.9 mg/kg; I 0.6 mg/kg; Co 0.2 mg/kg; Se 0.2 mg/kg; Vit A 30,000 IU/kg; Vit D_3_ 7500 IU/kg; Vit E 40 IU/kg; Vit K_3_ 4 mg/kg; B_1_ 6.99 mg/kg; B_2_ 8 mg/kg; Niacin 40 mg/kg; Pantothenic acid 30 mg/kg; Vit B_6_ 10 mg/kg; Folic acid 3.10 mg/kg; Biotin 0.39 mg/kg; Vit B_12_ 100 µg/kg; Choline 500 mg/kg; Lys 1.73%; Met 0.35%; ^2^ Mineral and vitamin composition: Ca 15.0%; P 4.55%; Na 0.28%; Cl 0.46%; K 9.53%; Mg 2.62%; S 2.23%; Fe 10.03 mg/kg; Cu 18.03 mg/kg; Mn 40.10 mg/kg; chelated Zn 55.14 mg/kg; I 0.80 mg/kg; Co 0.60 mg/kg; Se 0.63 mg/kg; Vit A 8.14 IU/g; Vit D_3_ 1.11 IU/g; Vit E 40.10 mg/kg.

**Table 2:** Feed intake and performance of dairy calves supplemented with sodium butyrate, organic zinc, or their combination during the pre-weaning and weaning periods.

Variable	Treatments ^1^				SEM ^2^	*p*-Value		
	COM	SB	OZn	SBOZn		Treat	Time	Treat * Time
Total, g DM/d	0.925	0.945	0.912	0.862	0.086	0.886	<0.0001	0.304
Milk replacer, g DM/d	0.619	0.627	0.620	0.629	0.044	0.991	<0.0001	0.951
Starter, g DM/d	0.315	0.324	0.312	0.256	0.073	0.885	<0.0001	0.286
Water intake, L/d	2.82	3.10	2.89	2.22	0.273	0.148	<0.0001	0.936
Mean BW, kg								
Initial	44.25	42.91	44.21	43.82	1.237	0.854	-	-
Final	73.64	70.18	71.76	67.31	3.279	0.507	-	-
Average of the total period	56.17	52.91	55.52	54.75	2.399	0.656	-	-
Weight gain, g/d	0.487	0.470	0.485	0.412	0.057	0.520	<0.0001	0.313
Gain:feed ratio	0.507	0.428	0.457	0.502	0.048	0.579	0.481	0.724
Body measures, cm								
Withers height	78.40	77.56	78.11	78.04	0.474	0.579	<0.0001	0.925
Hip width	23.64	23.21	23.47	23.37	0.237	0.469	<0.0001	0.722
Length	64.89	63.56	64.2	63.37	0.772	0.126	<0.0001	0.326

^1^ CON: basal diet with no supplementation; SB, calves supplemented with 3 g/kg DM of sodium butyrate (ADIMIX^®^ EASY, Adisseo, Antony, France); OZn, calves supplemented with 262 mg/kg DM of organic zinc (B-TRAXIM^®^, ADM, Chicago, IL, USA); SBOZn, calves supplemented with 3 g/kg DM of sodium butyrate (ADIMIX^®^ EASY, Adisseo) and 262 mg/kg DM of organic zinc (B-TRAXIM^®^, ADM). ^2^ Standard error of the mean.

**Table 3:** Zinc balance of dairy calves supplemented with sodium butyrate, organic zinc, or their combination during the pre-weaning period.

Variable	Treatments ^1^				SEM ^2^	*p*-Value
	CON	SB	OZn	SBOZn		
Zn intake, mg/d	205.78 ^b^	206.20 ^b^	267.77 ^a^	273.23 ^a^	14.313	0.002
Zn excretion, mg/d						
Feces	76.69	70.49	73.85	76.69	20.395	0.995
Urine	3.96	3.74	2.66	1.63	1.329	0.545
Total Zn excreted	80.74	74.16	76.28	78.67	20.430	0.993
Absorption						
Apparent absorption, mg/d	126.90 ^b^	137.38 ^b^	193.36 ^a^	190.59 ^a^	16.463	0.019
Absorbed Zn, %	62.61	66.46	72.56	72.62	7.624	0.746
Excreted Zn, %	37.39	33.54	27.45	27.02	7.624	0.746
Retention						
Zn retention, mg/d	122.94 ^b^	133.71 ^b^	190.94 ^a^	188.91 ^a^	16.678	0.017

^a,b^ Means within rows with different superscripts differ significantly in Tukey’s significant difference test (*p* < 0.05). ^1^ CON: basal diet with no supplementation; SB, calves supplemented with 3 g/kg DM of sodium butyrate (ADIMIX^®^ EASY, Adisseo); OZn, calves supplemented with 262 mg/kg DM of organic zinc (B-TRAXIM^®^, ADM); SBOZn, calves supplemented with 3 g/kg DM of sodium butyrate (ADIMIX^®^ EASY, Adisseo) and 262 mg/kg DM of organic zinc (B-TRAXIM^®^, ADM). ^2^ Standard error of the mean.

**Table 4:** Diarrhea incidence and fecal score in dairy calves supplemented with sodium butyrate, organic zinc, or their combination during the pre-weaning and weaning periods.

Variable	Treatments ^1^				SEM ^2^	*p*-Value		
	CON	SB	OZn	SBOZn		Treat	Time	Treat * Time
Incidence of diarrhea, %	91.67	100	100	100	-	0.382	-	-
Fecal score	0.803	0.853	0.996	0.847	0.129	0.293	<0.0001	0.833

^1^ CON: basal diet with no supplementation; SB, calves supplemented with 3 g/kg DM of sodium butyrate (ADIMIX^®^ EASY, Adisseo); OZn, calves supplemented with 262 mg/kg DM of organic zinc (B-TRAXIM^®^, ADM); SBOZn, calves supplemented with 3 g/kg DM of sodium butyrate (ADIMIX^®^ EASY, Adisseo) and 262 mg/kg DM of organic zinc (B-TRAXIM^®^, ADM). ^2^ Standard error of the mean.

**Table 5:** Gene expression of tight junction proteins in the jejunum of dairy calves supplemented with sodium butyrate, organic zinc, or a combination of both.

Variable *	Treatments ^1^				SEM ^2^	*p*-Value
	CON	SB	OZn	SBOZn		
Claudine 1	0.000197	0.000199	0.000386	0.000299	0.000076	0.245
Claudine 4	0.002171	0.003666	0.005486	0.005243	0.001331	0.312
Occludin	0.001166	0.000696	0.001075	0.000885	0.000172	0.135
ZO-1	0.003845	0.002824	0.003701	0.005229	0.000739	0.178

* Arbitrary units. ^1^ CON: basal diet with no supplementation; SB, calves supplemented with 3 g/kg DM of sodium butyrate (ADIMIX^®^ EASY, Adisseo); OZn, calves supplemented with 262 mg/kg DM of organic zinc (B-TRAXIM^®^, ADM); SBOZn, calves supplemented with 3 g/kg DM of sodium butyrate (ADIMIX^®^ EASY, Adisseo) and 262 mg/kg DM of organic zinc (B-TRAXIM^®^, ADM). ^2^ Standard error of the mean.

**Table 6:** Serum zinc and copper concentrations in dairy calves supplemented with sodium butyrate, organic zinc, or their combination during the pre-weaning and weaning periods.

Variable	Treatments ^1^				SEM ^2^	*p*-Value		
	CON	SB	OZn	SBOZn		Treat	Time	Treat * Time
Zn, ppm	1.732 ^y^	1.617 ^y^	2.196 ^x^	2.032 ^xy^	0.244	0.071	<0.0001	0.648
Cu, ppm	0.633	0.635	0.540	0.661	0.059	0.256	<0.0001	0.950

^x,y^ Means within rows with different superscripts tended to differ significantly in Tukey’s significant difference test (*p* > 0.05 and <0.10). ^1^ CON: basal diet with no supplementation; SB, calves supplemented with 3 g/kg DM of sodium butyrate (ADIMIX^®^ EASY, Adisseo); OZn, calves supplemented with 262 mg/kg DM of organic zinc (B-TRAXIM^®^, ADM); SBOZn, calves supplemented with 3 g/kg DM of sodium butyrate (ADIMIX^®^ EASY, Adisseo) and 262 mg/kg DM of organic zinc (B-TRAXIM^®^, ADM). ^2^ Standart error of the mean.

**Table 7:** Serum concentrations of acute phase proteins, immunoglobulins, and oxidative stress markers in dairy calves during the weaning period.

Variable	Treatments ^1^				SEM ^2^	*p*-Value		
	COM	SB	OZn	SBOZn		Treat	Time	Treat * Time
Albumin, g/L	35.84	34.73	34.71	35.67	1.408	0.901	0.334	0.114
Ceruloplasmin, mg/dL	49.76 ^b^	63.85 ^ab^	46.55 ^b^	68.76 ^a^	4.985	0.015	0.044	0.326
Haptoglobin, mg/dL	43.16	38.48	51.41	30.57	7.074	0.300	0.006	0.936
Transferrin, mg/dL	331.54	327.45	293.77	316.53	16.23	0.407	0.001	0.242
IgA, mg/dL	133.05	159.24	125.46	138.34	22.63	0.425	0.018	0.642
IgG, g/L	7.87	7.62	5.52	9.15	1.048	0.108	0.011	0.208
SOD ^3^, U/mL	1.917 ^x^	1.983 ^x^	1.573 ^y^	1.580 ^y^	0.135	0.087	0.135	0.905
ASD ^4^, Trolox	0.915	1.086	0.835	1.165	0.151	0.431	<0.0001	0.219

^a,b^ Means within rows with different superscripts differ significantly in Tukey’s significant difference test (*p* < 0.05). ^x,y^ Means within rows with different superscripts tended to differ significantly in Tukey’s significant difference test (*p* > 0.05 and < 0.10). ^1^ CON: basal diet with no supplementation; SB, calves supplemented with 3 g/kg DM of sodium butyrate (ADIMIX^®^ EASY, Adisseo); OZn, calves supplemented with 262 mg/kg DM of organic zinc (B-TRAXIM^®^, ADM); SBOZn, calves supplemented with 3 g/kg DM of sodium butyrate (ADIMIX^®^ EASY, Adisseo) and 262 mg/kg DM of organic zinc (B-TRAXIM^®^, ADM). ^2^ Standard error of the mean. ^3^ Superoxide dismutase. ^4^ Total antioxidant activity.

## Data Availability

The raw data supporting the conclusions of this article will be made available by the authors on request.

## Supplementary Materials

The following supporting information can be downloaded at: https://www.mdpi.com/article/10.3390/ani16020230/s1, Figure S1. Fecal score of dairy calves supplemented with sodium butyrate, organic zinc, or their combination during the pre-weaning and weaning periods. Figure S2. Frequency of fecal scores 2 (a) and 3 (b) in dairy calves supplemented with sodium butyrate, organic zinc, or their combination during the pre-weaning and weaning periods.

## Abbreviations

The following abbreviations are used in this manuscript:

BW	Body weight
CP	Crude protein
DM	Dry matter
EE	Ether extract
NDF	Neutral detergent fiber
THI	Temperature–humidity index
