# Supplementary material for: Effects of Sodium Butyrate and Organic Zinc Supplementation on Performance, Mineral Metabolism, and Intestinal Health of Dairy Calves
Source: Animals (Basel). 2026 Jan 13;16(2):230. doi: 10.3390/ani16020230 (PMC12837404; doi:10.3390/ani16020230)
Supplement: Supplementary file 1 [file animals-16-00230-s001.zip › animals-3998210-supplementary.pdf]

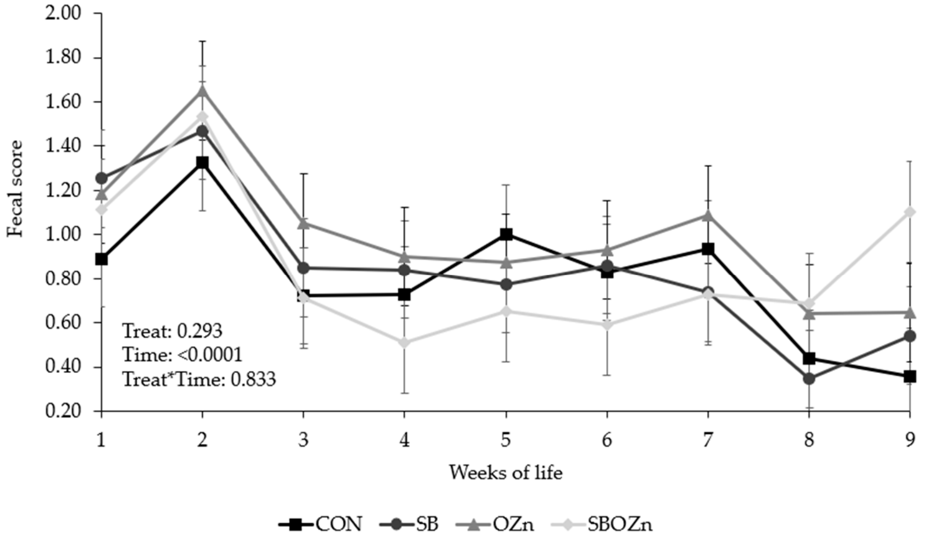

Figure S1. Fecal score of dairy calves supplemented with sodium butyrate, organic zinc, or their combination during the pre-weaning and weaning periods.
CON: basal diet without supplementation; SB: calves supplemented with 3 g/kg DM of sodium butyrate (ADIMIX® EASY, Adisseo); OZn: calves supplemented with 262 mg/kg DM of organic zinc (B-TRAXIM®, ADM); SBOZn: calves supplemented with 3 g/kg DM of sodium butyrate (ADIMIX® EASY, Adisseo) + 262 mg/kg DM of organic zinc (B-TRAXIM®, ADM). Error bars are SEM (standard error of the mean).

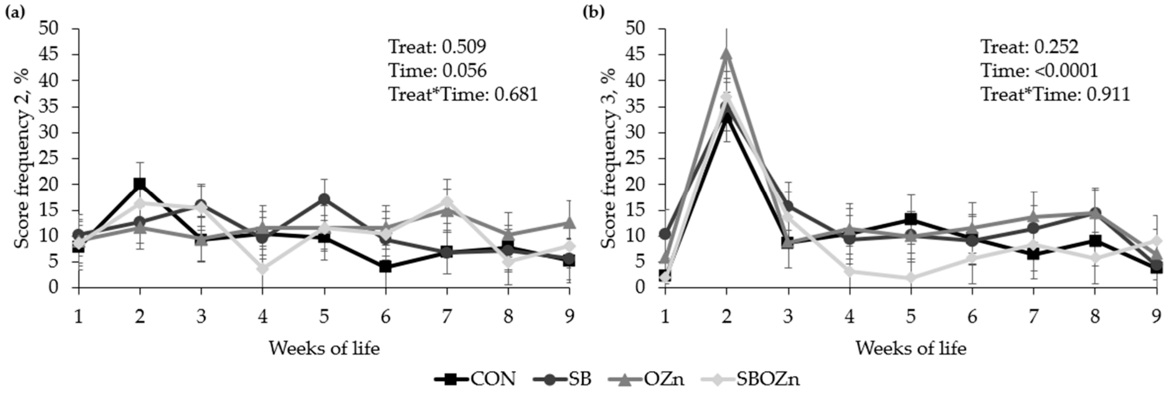

Figure S2. Frequency of fecal scores 2 (a) and 3 (b) in dairy calves supplemented with sodium butyrate, organic zinc, or their combination during the pre-weaning and weaning periods.
CON: basal diet without supplementation; SB: calves supplemented with 3 g/kg DM of sodium butyrate (ADIMIX® EASY, Adisseo); OZn: calves supplemented with 262 mg/kg DM of organic zinc (B-TRAXIM®, ADM); SBOZn: calves supplemented with 3 g/kg DM of sodium butyrate (ADIMIX® EASY, Adisseo) + 262 mg/kg DM of organic zinc (B-TRAXIM®, ADM). Error bars are SEM (standard error of the mean).
